# Adaptive approach for tracking movements of biological targets: application to robot-based intervention for prostate cancer

**DOI:** 10.3389/frobt.2024.1416662

**Published:** 2024-08-12

**Authors:** Abdeslem Smahi, Othman Lakhal, Taha Chettibi, Mario Sanz Lopez, David Pasquier, Rochdi Merzouki

**Affiliations:** ^1^ CRIStAL, CNRS-UMR 9189, University of Lille, Villeneuve d’Ascq, France; ^2^ Department of Mechanical Engineering, Blida-1 University, Blida, Algeria; ^3^ Academic Department of Radiation Oncology, Centre O. Lambret, Lille, France

**Keywords:** medical robot, adaptive optimisation, prediction, path planning, artificial intelligence

## Abstract

**Introduction:**

In this paper, we introduce an advanced robotic system integrated with an adaptive optimization algorithm, tailored for Brachytherapy in prostate cancer treatment. The primary innovation of the system is the algorithm itself, designed to dynamically adjust needle trajectories in response to the real-time movements of the prostate gland during the local intervention.

**Methods:**

The system employs real-time position data extracted from Magnetic Resonance Imaging (MRI) to ensure precise targeting of the prostate, adapting to its constant motion and deformation. This precision is crucial in Brachytherapy, where the accurate placement of radioactive seeds directly impacts the efficacy of the treatment and minimizes damage to surrounding safe tissues.

**Results:**

Our results demonstrate a marked improvement in the accuracy of radiation seed placement, directly correlating to more effective radiation delivery. The adaptive nature of the algorithm significantly reduces the number of needle insertions, leading to a less invasive treatment experience for patients. This reduction in needle insertions also contributes to lower risks of infection and shorter recovery times.

**Discussion:**

This novel robotic system, enhanced by the adaptive optimization algorithm, improves the coverage of targets reached by a traditional combinatorial approach by approximately 15% with fewer required needles. The improved precision and reduced invasiveness highlight the potential of this system to enhance the overall effectiveness and patient experience in prostate cancer Brachytherapy.

## 1 Introduction

With approximately 1.5 million newly diagnosed cases and 397,000 fatalities reported globally, prostate cancer stands as the second most common cancer and ranks as the fifth principal contributor to cancer-related mortality in the male population for the year 2022 (Bray et al., 2024). Its treatment typically surgery, external radiotherapy, or brachytherapy (BT), which are validated by the High Authorities for Health (Dhaliwal et al., 2021a) and follow common guidelines. The guidelines, for giving radiotherapy and brachytherapy are thoroughly explained in established recommendations. Specifically the (National Comprehensive Cancer Network, 2024) and the guide for brachytherapy in Henry et al. (2022). These guidelines outline the criteria, for selecting patients the best treatment dosages and procedural methods to ensure that treatment is effective and customized to each patients situation.

**FIGURE 1:**
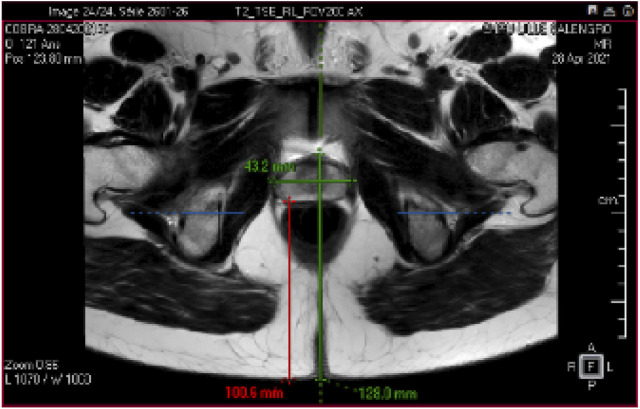
Prostate MR Image: The prostate gland is a walnut-sized organ located just below the bladder and in front of the rectum in males. An example of the size of an adult and healthy prostate is 43.2 mm length and 27.4 mm width. Understanding the 3D anatomy of the prostate is crucial for diagnosing and treating conditions such as benign prostatic hyperplasia (BPH) and prostate cancer.

These treatments are integral to a multidisciplinary management strategy that includes urologists, radiation oncologists, and the patient, who plays a crucial role in decision-making. Surgical approaches typically involve the complete removal of the prostate, while external radiotherapy requires delivering 70 Gy (Gy) across 35 sessions using a particle accelerator. Brachytherapy presents a more patient-tolerant option, utilizing either low-dose (LDR) or high-dose (HDR) ionizing radiation to target the affected area, typically guided by trans-rectal ultrasound (TRUS) imagery. This method, known as TRUS-BT, has traditionally been preferred for guiding the brachytherapy process. However, TRUS-BT faces several challenges which include primarly the rigid grid limited insertion angles (Belarouci et al., 2022) which has been improved by Lakhal et al. (2023) by proposing a new type of grid (See Figure 1).

The primary focus of this paper is to optimize these minimally invasive procedures by reducing the number of needles inserted into the organ. This approach aims to decrease patient discomfort and enhance the overall efficiency of the treatment, addressing some of the inherent limitations of the current methodologies.

### 1.1 Organ motion in clinical settings

Organ motion (Like the prostate motion in Figure 1) in clinical settings plays a crucial role in radiotherapy treatment planning and delivery. Studies have shown that organ motion, particularly in the abdomen and thorax, can impact radiation dose distribution (Kavak et al., 2022; Ng et al., 2023). Techniques like real-time MRI imaging and dynamic MR-guided radiotherapy have been developed to address and correct for organ motion in real-time, ensuring optimal treatment doses with minimal toxicity to nearby organs at risk (Senneville et al., 2015; Kannan et al., 2017). Similarly, the accuracy of motion tracking in the liver using 4D ultrasound has been validated, offering potential for interventions on moving abdominal organs (Vijayan et al., 2014). Lastly, adaptive compensation for subject motion in real-time during MRI scans has been achieved through the use of object orientation markers, allowing for continuous correction of scan planes (Ernst et al., 2019). These advancements collectively underscore the critical importance and ongoing efforts to accurately account for and manage organ motion in clinical settings. Consequently, the integration of advanced AI methods for predicting organ motion before it occurs has recently become a focal point in clinical settings. These predictive technologies are designed to further refine treatment approaches, ensuring even higher precision and improved outcomes for patients.

### 1.2 Organ motion prediction techniques

In treatments for prostate cancer, the Prostate Motion AI-based Prediction Model focuses on translational and rotational movements of the prostate, overlooking deformations. Advanced time series forecasting models are crucial for precise organ movement tracking during such medical procedures.

Convolutional Neural Networks (CNNs) excel in detecting spatial patterns (Bai et al., 2018; Ismail and Sengur, 2021), while Long Short-Term Memory (LSTM) networks are adept at capturing extended sequences (Chauhan and Vig, 2015; Yang et al., 2023). Graph-based models effectively manage topological data (Liao et al., 2023; Wang et al., 2023; Xiao et al., 2023), and hypergraph-based models capture complex relationships, useful in varied domains like stock market predictions (Huynh et al., 2022).

Dynamic MRI techniques facilitate real-time motion tracking, addressing motion artifacts in procedures such as PET scans (Frohwein et al., 2019; Li et al., 2019; Purushotham et al., 2019; Bengs et al., 2023). Deep learning models, renowned for intricate pattern recognition, are increasingly employed for motion prediction in healthcare, promising enhanced diagnostic accuracy (Krebs et al., 2019; Morid et al., 2021).

The ability to predict organs motion is vital for optimizing minimally invasive interventions, enabling clinicians to perform procedures with greater accuracy and reduced risk. By integrating such advanced motion prediction models, healthcare professionals can significantly enhance the operational precision required in these sensitive clinical environments.

### 1.3 Optimization in local interventions

High precision and a low level of patient trauma are necessary for minimally invasive procedures, particularly in targeted therapies like prostate cancer treatment. The physical and algorithmic approaches to optimization tactics in these procedures can be roughly classified as improving the effectiveness and efficiency of treatments.

**FIGURE 2:**
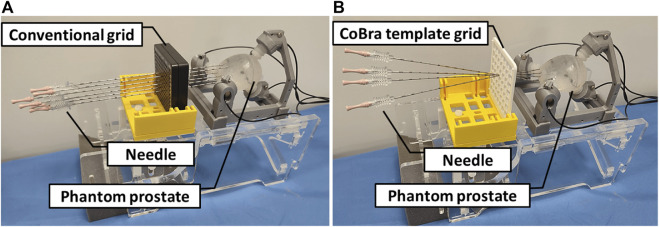
**(A)** Conventional parallel needle insertions from multiple entry points: This can cause oedema and trauma to patients due to multiple insertion points; **(B)** Optimal oblique insertions point prostate targeting: One needle can be inserted from one entry point with different orientations for optimal coverage of the target volume, which means a faster and less traumatic treatment for the patients.

Physical optimization in minimally invasive procedures primarily involves enhancing the method and tooling used for interventions. Figure 2 represents the suggestion of Dhaliwal et al. (2021b) to use oblique needle insertion techniques instead of a traditional straight needle insertions which minimise the number of insertions drastically since one needle is used to reach a larger area and does not impact the medicament delivery (Su et al., 2019; Li et al., 2022) introduced manipulability optimization control for a 7-DoF robot manipulator in Robot-Assisted Minimally Invasive Surgery, ensuring Remote Center of Motion (RCM) and improved manipulability.

As for Algorithmic approaches, various studies have explored the application of optimization algorithms in different aspects of minimally invasive surgery (Ruijters, 2021). proposed evolutionary computing strategies like APSO and OBDE to optimize electromagnetic sensor measurements, significantly reducing tracking errors in surgical navigation. Surgical planning and training have also benefited from algorithmic optimization. Hyuck et al. (2020) have introduced a computer-based surgery optimization method using genetic algorithms to derive optimal surgical procedures. Additionally, Lakhal et al. (2023) introduced a combinatorial optimization problem approach for efficient path planning, which is technically advantageous for making oblique insertions and clinically beneficial for reducing harm to patients.

### 1.4 Main contributions

**FIGURE 3:**
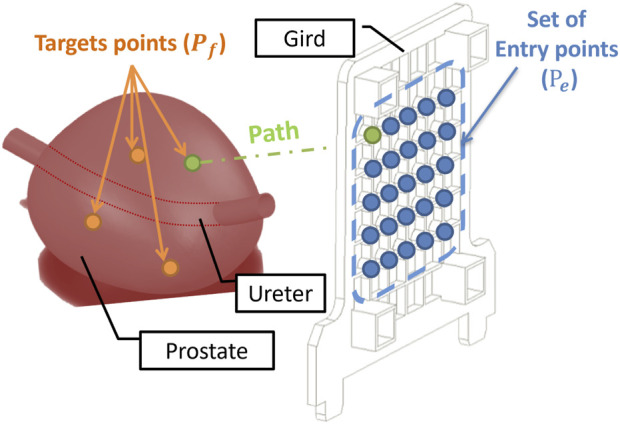
Optimisation Problem: The blue points describe the set of entry points of the grid installed along the perineal skin. The orange points represent the target tumours in the prostate. Each needle path (green) is defined by a point in the target and an entry point.

**FIGURE 4:**
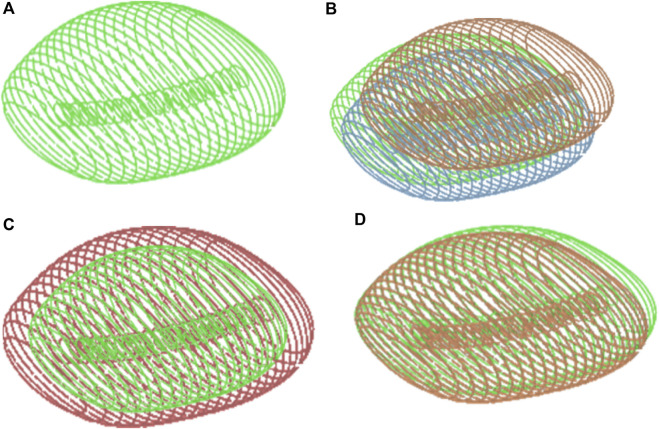
**(A)** Prostate 3D Model. The Prostate gland surrounds the urethra, which is the tube that carries urine from the bladder out of the body. In three-dimensional anatomical terms, the prostate gland has a roughly spherical shape with a central indentation where the urethra passes through. **(B)** Prostate movements: The movement of the prostate involves dynamic shifts in its position and shape within the pelvis. It moves in response to physiological processes such as respiration, bladder filling and emptying and pelvic floor muscle activity. **(C)** Prostate Inflammation: It can affect the 3D geometric movement of the gland by causing swelling and enlargement. This can lead to changes in the shape and structure of the prostate, potentially impacting its surrounding structures such as the urethra and bladder. Inflammation can distort the normally smooth contours of the prostate, leading to irregularities in its shape and size. z**(D)** Prostate Deformation: After a local intervention such as surgery or radiation therapy for prostate cancer, the prostate gland may undergo deformation. This deformation can result from tissue removal, scarring, or changes in the structure of the gland due to the intervention. As a result, the shape, size, and position of the prostate may be altered from its original state. Illustrations of Prostate volume configurations: **(A)** Prostate 3D Model; **(B)** Prostate movements; **(C)** Prostate Inflammation; **(D)** Prostate Deformation.

Building on the previous discussions regarding organ motion and the optimization of minimally invasive procedures, we extend the research initiated by Lakhal et al. (2023) by implementing the following methodologies:

• Developing a predictive model for organ motion using a deep learning framework that combines Long Short-Term Memory (LSTM) networks and Convolutional Neural Networks (CNNs).
• Enhancing the approach to minimally invasive prostate interventions by incorporating predicted organ movements into the procedure planning (See Figures 3, 4)

These initiatives aim to refine the precision and efficacy of treatments by anticipating and adjusting for organ motion, thereby improving the overall outcomes of minimally invasive procedures.

## 2 Materials and methods

**FIGURE 5:**
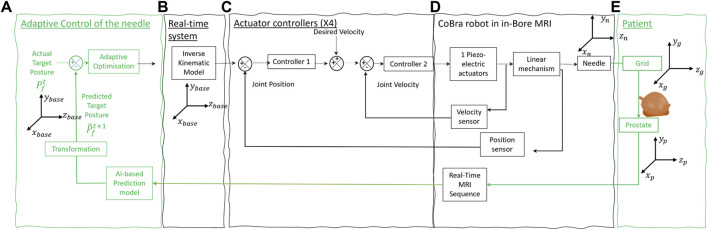
Block diagram of the patient + The control architecture of CoBra robot is composed of four main parts. **(A)** Represents the main contribution of the paper using AI-based prediction model and adaptive optimisation algorithm for adaptive control of the needle. **(B–D)** represent modelling, control and instrumentation respectively and they have been the subject of previous studies (Dhaliwal et al., 2019; Dhaliwal et al., 2021a; Belarouci et al., 2022). **(E)** Represents the organ in the patient body and its interaction with the system.

This section describes the CoBra robotic system tools and methodologies in precision needle control for precision needle control in medical procedures. The system in Figure 5 is composed of AI-based prediction, adaptive optimization, system modeling, control mechanisms, and instrumentation. All of them are vital to enhance the precision and effectiveness of needle navigation.

Our main focus is on segment (a) of the system which follows a novel approach by integrating an AI-based prediction model with an adaptive optimization algorithm. Its aim is to enhance the way the needle is controlled through adapting its path in real-time in response to changes inside the patient’s body. This segment is the main contribution of the present work, building upon previously published foundational work in modeling by Belarouci et al. (2022), control (Dhaliwal et al., 2019), and instrumentation (Dhaliwal et al., 2021a).

The global system in Figure 5 uses four different coordinates, the coordinate systems $\left(x_{\text{base}},y_{\text{base}},z_{\text{base}}\right)$ , $\left(x_{n},y_{n},z_{n}\right)$ , $\left(x_{g},y_{g},z_{g}\right)$ , and $\left(x_{p},y_{p},z_{p}\right)$ define the positions and orientations of various components. $\left(x_{\text{base}},y_{\text{base}},z_{\text{base}}\right)$ is typically considered as the global origin for the system, serving as the foundational reference for all other coordinates. $\left(x_{n},y_{n},z_{n}\right)$ describes the needle’s position and orientation. $\left(x_{g},y_{g},z_{g}\right)$ specifies the grid’s alignment and position, ensuring that the needle’s path through the grid is correctly aligned for precise insertion. $\left(x_{p},y_{p},z_{p}\right)$ , the coordinates for the prostate or target area, detail the target’s position and orientation.

The AI model forecasts future movements of the target organ, essential for guiding the needle precisely to the right area despite the motion of organs. The adaptive optimization algorithm utilizes these predictions for refining the needle trajectory in a way that ensures the needle is always pointed toward the target.

This section covers in detail the operational principles of segment (a), addressing how the AI model predicts the movement of organs and how the optimization algorithm adapts the path of the needle.

### 2.1 MR-robot for prostate intervention

The prostate organ is a small, walnut-sized gland situated below the bladder and in front of the rectum in males. The normal sized prostate is typically about 4 cm wide, 3 cm high, and 2 cm thick, though its size can vary slightly between individuals.

When the procedure is less invasive, such as in a biopsy or brachytherapy, it is possible to approach the prostate through the perineal or rectal route. Regarding the CoBra robot application (Dhaliwal et al., 2019), the desired entry point is the perineum. Laparoscopic surgery is performed through multiple small incisions made in the abdomen of the patient, who is positioned in the lithotomy position. The lithotomy position involves the patient lying on their back, with the legs drawn up at the hip and knee joints and the feet resting on stirrups. This position exposes the perineal area well, assisting the clinician in conveniently reaching the prostate.

The perineal approach entails the use of a needle inserted through the skin of the perineum and into the prostate gland with the help of imaging techniques such as MRI for the CoBra robot. This approach reduces the impact on other tissues and results in a brief healing period compared to other surgical procedures.

The CoBra robot is a parallel robot with 5 degrees of freedom (DoF) designed for interventions under MRI. The concept allows for oblique insertion with minimal access points through the perineum to target multiple lesion sites. To ensure precise intervention, the system is equipped with absolute encoders, including the *LAK14-Heidenhain* from Traunreut and the *Numerik-Jena* from Jena, offering a scale resolution of 1.25 μm. This high precision enables accurate guidance and needle insertion when interacting with tissues during medical procedures. The use of these absolute encoders for each of the five degrees of freedom movements is crucial to ensure closed-loop position control. Absolute encoders immediately provide position information upon power-up, ensuring both precision and safety. Interrupting a procedure due to loss of position information is unacceptable in a clinical environment. Therefore, maintaining backup power is imperative to prevent any abrupt stops or power failures.

**FIGURE 6:**
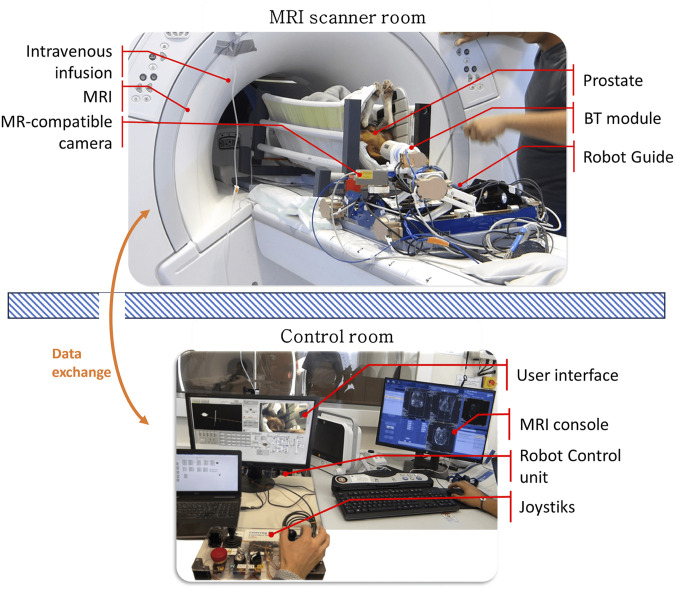
The CoBra system combines multiple components, including a 5-DoF parallel robot as a needle guide, a BT module, an MRI scanner, and an MR camera. The patient in this case an animal (dog) is positioned in a lithotomy position and given sedation while being supported. The CoBra system’s architecture allows for tele-operation using a joystick from the control room, utilizing the MRI console and a graphical interface for robot control.

The needle-based intervention robot-guide can operate inside the MRI scanner tunnel, in the workspace located under the patient’s legs in a lithotomy position. This concept is compatible with large-diameter 3T MRI scanners, featuring a 70 cm diameter bore. Access to the prostate is achieved through a transperineal (TP) approach in the lithotomy position rather than a lateral decubitus position. To facilitate operator access and provide real-time visualization for teleoperation control and patient monitoring, the CoBra system is equipped with an MRI-compatible camera (Figure 6).

The main reasons for using the CoBra robot instead of a single arm (?) for minimally invasive intervention on the prostate are:

1. Restricted Workspace Adaptability: The CoBra robot can move and reach the perineal skin in a 20 cm by 15 cm opening with a 15 cm penetration depth in the MRI scanner. A parallel kinematic chain of the CoBra robot is more transportable, stable, and precise compared to an open kinematic chain of a single serial arm.
2. MRI Compatibility: Current single serial arm designs are unsuitable for use inside the MRI scanner where the magnetic field affects the operation of the joint actuators. The CoBra robot is designed to clinically meet placement, control accuracy, and MR-compatibility specifications, ensuring that it can operate effectively within the MRI environment.

In summary, the CoBra robot’s design addresses the limitations of single arm minimally invasive surgical robots, particularly in the context of MRI-guided prostate interventions. Its precision, stability, and compatibility with MRI technology make it a superior choice for clinicians aiming to perform minimally invasive procedures with high accuracy and minimal patient recovery time.

### 2.2 Image-based robot registration and robot kinematics

**FIGURE 7:**
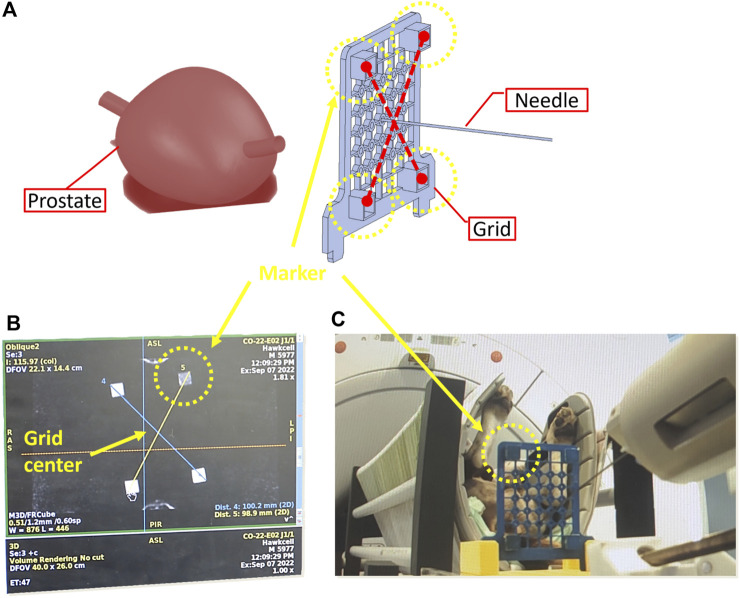
Calibration phase to determine the coordinates of the needle tip in the MRI frame. **(A)** 3D reconstruction of pre-operative scans **(B)** needle tip coordinates based on MRI imaging **(C)** Placement of needle in the centre of the grid.

**FIGURE 8:**
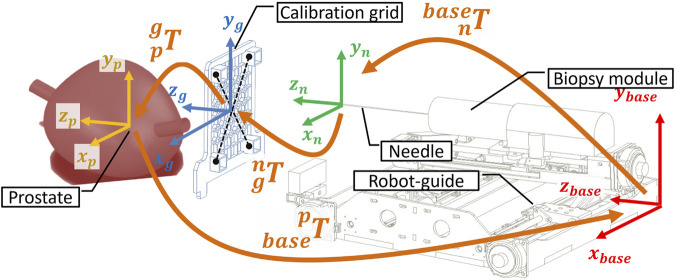
Kinematic transformation chain to calibrate the robot in the MRI coordinate system based on the imaging of the dog lying inside the scanner bore in lithotomy position.

A calibration procedure, referred to as registration, is conducted using pre-operative MRI scans to align the robot’s reference frame with the prostate’s coordinate system (see Figures 7, 8).

To commence the registration process, an MRI-compatible grid is positioned against the perineum using a suitable support structure (Figure 7A). This grid features four pockets filled with agar-agar, which are visible under MRI imaging. Subsequently, the needle’s tip is positioned at the midpoint of the grid (Figure 7C). Pre-operative scanning facilitates the identification of various frames attached to the robot (Figure 7B), the needle, and the prostate gland, as illustrated in Figure 8.

The frames $R_{\mathrm{base}}$ , $R_{n}$ , $R_{g}$ , and $R_{p}$ are respectively associated with the base of the robot-guide, the needle tip, the center of the grid, and the center of the prostate gland, as depicted in Figure 8. The transformations ${base}^{p}T$ , $n^{\mathrm{base}}T$ , and Tpg are derived from MRI imaging and forward kinematic models, enabling the calculation of the global transformation using Equation 1. Once calibration is accomplished, the inverse kinematic model becomes operational.

$${base}^{p}T={base}^{n}T_{n}^{g}T_{g}^{p}T$$ (1)

### 2.3 Adaptive MR-based control of the CoBra needle guide robot

In this study, we will focus on the adaptive control system part in Figure 5A for needle path planning represents a subsystem within a larger therapeutic framework. This system is responsible for the real-time adjustment of needle trajectories during brachytherapy, ensuring high precision in targeting and treatment delivery. The aspects related to the instrumentation Figure 5D, modelling Figure 5B, and control Figure 5C of the Cobra robot have been the subject of further developments and previous studies (Dhaliwal et al., 2019; Belarouci et al., 2022) and Dhaliwal et al. (2021a).

This subsystem includes includes two main components:

• AI-based Prediction Model: At the foundation lies an AI-based prediction model that analyzes real-time organ motion (See Figure 2) that can be extracted from MRI data to anticipate the movement of the prostate. The dataset of positions was extracted directly from the MRI machine itself, which has the capability to segment the prostate and extract its posture but in cases where the MRI machine does not have the capability to perform this segmentation automatically, various methods can be applied to the image, such as those detailed in (Comelli et al., 2021). This model uses a prediction horizon of the next time step, which corresponds to $\frac{1}{r}s$ given the MRI image rate of r images per second (in our case r=8 ). This short-term horizon allows for precise real-time adjustments by forecasting the prostate’s immediate future position.
• Adaptive Optimisation: Leveraging the predictive model’s output, the adaptive optimisation module calculates the optimal needle insertion points. By doing so, it ensures efficient coverage of the cancerous area while minimising tissue damage and the number of needle insertions.

This section contextualizes the adaptive control system’s role within the complex network of processes and technologies involved in it, highlighting its critical function in enhancing treatment outcomes.

#### 2.3.1 Study hypotheses

Throughout the course of this study, several key hypotheses have been posited to guide the development of the predictive model and optimization framework. These are enumerated as follows:

1. Prostate Rigidity: It is postulated that the prostate is a rigid body during the brachytherapy procedure. This assumption underpins the algorithmic management of all movements—translational and rotational—across the six degrees of freedom characteristic of rigid body dynamics. The notion of rigidity simplifies the complex nature of prostate motion, streamlining predictive modeling by focusing on the essential aspects of spatial maneuverability without deformation complexities:

• Translation along the x, y, and *z*-axes.

• Rotation about the x, y, and *z*-axes.
2. Needle Interaction: We assume the influence of the brachytherapy needle on prostate motion is minimal. Mathematically, this is represented as:

Δp≈0,Δθ≈0 (2)

where Δp denotes the change in the prostate’s position vector and Δθ denotes the change in the prostate’s orientation vector due to the needle insertion.

These hypotheses are foundational to the design of our adaptive optimization algorithm and the AI-based predictive model. They allow for a more tractable problem space and a focused approach to enhancing the precision and efficacy of brachytherapy (or biopsy).

#### 2.3.2 Prostate motion AI-based prediction model

In brachytherapy for prostate cancer, the Prostate Motion AI-based Prediction Model focuses on translational and rotational movements of the prostate, overlooking deformations. Advanced time series forecasting models are crucial for precise organ movement tracking during such medical procedures.

Using a dataset of real motion data from 162 patients introduced in (Taillez et al., 2021) (the patients accepted the use of the motion for scientific research), with each patient monitored over five sessions (see Figure 10), the model is designed to capture the stochastic nature of prostate movement during therapeutic procedures.

**FIGURE 9:**
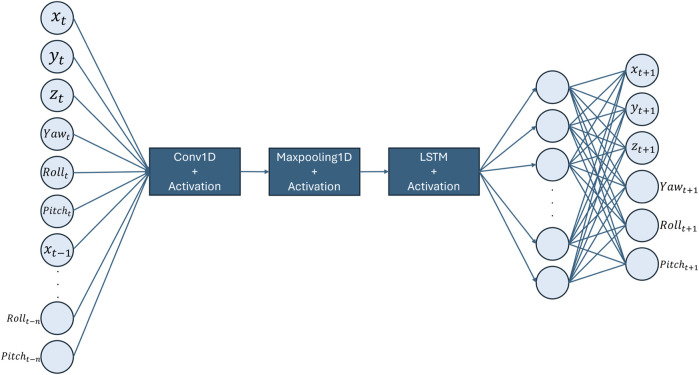
CNN-LSTM motion prediction model.

The architecture of choice is a Convolutional Neural Network-Long Short-Term Memory (CNN-LSTM) model shown in Figure 9. This method harnesses the spatial feature extraction capabilities of CNNs from MRI sequences and the temporal pattern recognition prowess of LSTMs. CNNs excel in identifying hierarchical patterns in spatial data, making them ideal for interpreting medical images such as MRI scans. On the other hand, LSTMs are adept at capturing long-term dependencies in sequential data, allowing the model to understand the temporal dynamics of organ movement (Keerthana et al., 2023).

The spatial-temporal data, encapsulating the 3D prostate posture at time t , is denoted as $X_{t}$ . Accurate real-time tracking of the prostate’s movement is crucial in precision medicine. Recent advancements in deep learning, particularly CNNs, have shown promise in enhancing the precision of real-time image-guided therapies for prostate cancer, indicating the potential of these technologies in improving treatment outcomes (Chrystall et al., 2023).

The predictive model is formally expressed as:

$$X_{t+1}=\text{CNN}-\text{LSTM}\left(X_{t},X_{t-1},\ldots ,X_{t-n}\right)$$ (3)

where $X_{t}=\left\{x_{t},y_{t},z_{t},roll_{t},pitch_{t},yaw_{t}\right\}$ represents the prostate posture at time t and n represents the number of previous time steps taken into account for predicting the next prostate’s posture.

The training process involves the minimization of the Mean Squared Error (MSE) between the predicted and actual positions and orientations of the prostate’s center of gravity:

$$\min\text{MSE}=\min\frac{1}{N}\overset{N}{\underset{i=1}{\sum }}{\left(X_{t+1}^{\left(i\right)}-{\hat{X}}_{t+1}^{\left(i\right)}\right)}^{2}$$ (4)

where ${\hat{X}}_{t+1}^{\left(i\right)}$ represents the predicted position and orientation, $X_{t+1}^{\left(i\right)}$ represents the true position and orientation at time t+1 and N denotes the total number of observations in the dataset used for training the model. This optimization criterion ensures that the model provides the most accurate real-time predictions of prostate motion, which are critical for adjusting the needle trajectory during the brachytherapy procedure, thereby enhancing the precision and safety of the treatment.

#### 2.3.3 Path planning adaptive optimisation

Traditional biopsy (or brachytherapy) methods use a grid allowing only parallel needle insertions, often necessitating multiple insertions for comprehensive prostate coverage as shown in Figure 3A. However, this fails to consider the prostate’s dynamic nature and potential movement. To overcome this, the MPEP (Minimum Perineum Entry Points) approach introduced in Lakhal et al. (2023) minimizes insertions while maximizing tumor targeting. The CoBra template grid (CTG) guides needle paths under varying angles, adapting to prostate motion for more accurate targeting.

This study builds on the MPEP concept, factoring in dynamic prostate positioning to refine the adaptive optimization process. The aim is to optimally reduce needle insertions while ensuring precise targeting, considering prostate motion.

As illustrated in Figure 4, each needle trajectory is characterized by a target tumor point and an entry point. Here, the term “path” refers to the needle’s trajectory through the human body, originating from an entry point and terminating at a target point. Consequently, a complete treatment comprises N paths to address all N designated target tumors within the prostate. The problem can be mathematically formulated in the following manner.

Let $I_{f}$ represent a finite set of N target points $P_{f}$ , and $I_{e}$ denote a finite set of $N_{e}$ entry points $P_{e}$ , chosen from the grid. A treatment requires N paths to address N target tumors in the prostate, meaning a viable solution is a finite set of N feasible paths $P_{e}P_{f}$ such that $P_{f}\in I_{f}$ and $P_{e}\in I_{e}$ . It is important to note that a single entry point may be used to reach multiple target points. The challenge lies in identifying the most efficient combination of N feasible paths $P_{e}P_{f}$ that minimizes the number of entry points used, thereby also minimizing the robot’s travel distance within the prostate during treatment while taking into consideration the movements of the prostate.

The adaptive control segment, depicted in green in Figure 5, is based on an Artificial Intelligence (AI)-driven prediction model that utilizes real-time Magnetic Resonance Imaging (MRI) data. This innovative model forecasts the future position of the prostate, denoted as $P_{\mathrm{future}}$ , by analyzing both the current and past observed positions, symbolized as $P_{\mathrm{current}}$ and $P_{\mathrm{past}}$ , respectively:

$$P_{f}^{\left(t+1\right)}=f\left(P_{f}^{\left(t\right)},P_{f}^{\left(t-1\right)},\ldots ,P_{f}^{\left(t-k\right)}\right)$$ (5)

where $P_{f}^{\left(t+1\right)}$ represents the predicted future position of the prostate at time t+1 , f is the function implemented by the AI-based model encapsulating the dynamics of prostate motion, and $P_{f}^{\left(t-k\right)},\ldots ,P_{f}^{\left(t\right)}$ are the observed positions from time t−k to t , with k being the number of time steps considered for prediction.

Given a set of target points $\left\{P_{f_{1}},P_{f_{2}},\ldots ,P_{f_{n}}\right\}$ and a set of entry points $\left\{P_{e_{1}},P_{e_{2}},\ldots ,P_{e_{m}}\right\}$ , where n and m indicate the number of target and entry points respectively, the terms in the cost function are defined as follows:

minC=αR+βD−λE (6)

where:

• The term R represents the total distance between each entry point and its corresponding target point. For each path i from an entry point $P_{e_{i}}$ to a target point $P_{f_{i}}$ , the distance $d_{i}$ is calculated. The sum of all such distances is R :

$$R=\overset{N}{\underset{i=1}{\sum }}d\left(P_{e_{i}},P_{f_{i}}\right)$$ (7)

• The term D is the sum of the total distance from the predicted target positions to the entry point. For a predicted target position ${\hat{P}}_{f_{i}}$ and an entry point $P_{e_{i}}$ :

$$D=\overset{N}{\underset{i=1}{\sum }}d\left({\hat{P}}_{f_{i}},P_{e_{i}}\right)$$ (8)

• The term E is the number of selected entry points used in the procedure. If an entry point $P_{e_{j}}$ is used for any target point $P_{f_{i}}$ , it is included in the count:

$$E=|\left\{P_{e_{j}}:\exists P_{f_{i}}\text{ such that }P_{e_{j}}\text{ is used to reach }P_{f_{i}}\right\}|$$ (9)

• α weights the total distance R , A higher α value emphasizes the need to reduce the overall travel distance during the procedure, which can be crucial for minimizing tissue damage.
• β modulates the impact of the sum of distances from the entry points D to the predicted target positions. A higher β emphasizes minimizing these distances, thereby tailoring the prediction to favor shorter paths from the entry points to the predicted target positions.
• λ is introduced to equalize the scale between quantities that inherently differ in nature, such as a count of entry points (E) and the other distances.

The optimization problem formulated in this study is inherently nonlinear and constrained, encompassing both continuous and combinatorial aspects. The nonlinear nature arises from the complex dynamics of prostate movement and the associated prediction model, which influence the path optimization. It is a constrained problem, as it requires adherence to specific clinical and anatomical limitations, such as the feasible insertion points and paths that needles can take.

Furthermore, the objective function, integrating distance metrics and entry point counts, introduces a combinatorial challenge in selecting the optimal set of entry and target points to minimize overall procedure impact while maximizing accuracy. This combination of factors categorizes the optimization as a complex, multi-faceted challenge suitable for advanced computational techniques and heuristic approaches to find effective solutions.

#### 2.3.4 Dynamic entry points optimization and selection algorithm

Algorithm 1 shows the steps for the adaptive optimisation procedure, it is designed to dynamically optimize the selection of entry points for real-time therapeutic interventions. This method leverages predictive modeling combined with a cost-efficient search strategy, ensuring both high accuracy and operational efficiency in clinical settings.

Algorithm 1 Dynamic Entry Point Selection based on Motion Prediction and Cost Optimization.

1:**procedure** OptimizeEntryPoint ( $\left\{P_{f_{1}},P_{f_{2}},\ldots ,P_{f_{n}}\right\}$ , $\left\{P_{e_{1}},P_{e_{2}},\ldots ,P_{e_{m}}\right\}$ , f , α , β , λ )
2:    Initialize time t=0

3:    Load predictive model f

4:    Initialize active targets list with $\left\{P_{f_{1}},P_{f_{2}},\ldots ,P_{f_{n}}\right\}$

5:    **while** active targets not empty **do**
6:      Use f to predict $P_{f}^{\left(t+1\right)}$ for each active target point
7:      Initialize best entry point as null
8:      Initialize minimum cost to ∞

9:      **for** each entry point $P_{e_{j}}$ **do**
10:        Initialize distance cost $R_{j}=0$

11:        Initialize deviation cost $D_{j}=0$

12:        Initialize a list of reachable target points from $P_{e_{j}}$

13:        **for** each target point $P_{f_{i}}^{\left(t+1\right)}$ in active targets **do**
14:           **if** $P_{f_{i}}^{\left(t+1\right)}$ is reachable from $P_{e_{j}}$ **then**
15:              Add $P_{f_{i}}^{\left(t+1\right)}$ to reachable list
16:              Compute distance cost for this path $R_{ij}$

17:              Compute deviation cost for this path $D_{ij}$

18:              Add $R_{ij}$ to $R_{j}$

19:              Add $D_{ij}$ to $D_{j}$

20:           **end if**
21:        **end for**
22:        Compute entry point usage cost $E_{j}=\lambda \times \text{number of reachable targets by }P_{e_{j}}$

23:        Compute total cost for $P_{e_{j}}$ as $C_{j}=\alpha R_{j}+\beta D_{j}+E_{j}$

24:        **if** $C_{j}<\text{minimum cost}$ **then**
25:          Update minimum cost to $C_{j}$

26:          Update best entry point to $P_{e_{j}}$

27:        **end if**
28:      **end for**
29:      Implement path from best entry point to its reachable target points for t+1

30:      Remove the reached target points from active targets
31:      Increment t

32:      Update f with latest MRI data
33:    **end while**
34: **end procedure**

The process begins with an initialization phase where the predictive model f is loaded, and a list of active target points is prepared based on the initial set of target points. The model f is responsible for predicting the positions of target points at the next time step t+1 , using posture data. This prediction is crucial as it allows the algorithm to adjust its strategy based on the anticipated motion of the prostate, which can vary significantly from one patient to another.

After the initialization, the core of the algorithm operates in a loop that continues until all target points have been successfully reached or the procedure is deemed complete. Within this loop, the algorithm performs a greedy search to determine the most cost-effective entry point for each prediction cycle. For each entry point $P_{e_{j}}$ , the algorithm compute the associated cost. Once these costs are computed, the entry point with the lowest total cost is selected, and the corresponding path is implemented.

After selecting and implementing the best path for the current time step, the algorithm removes the reached target points from the list of active targets. The time variable t is then incremented, and the predictive model f is updated with new posture data from the MRI images to reflect the latest anatomical positioning.

##### 2.3.4.1 Complexity analysis

The iterative approach detailed in Algorithm 1 is designed to dynamically select optimal entry points in real-time therapeutic interventions. The timing of the algorithm is primarily a function of the number of target points $\left\{P_{f_{1}},P_{f_{2}},\ldots ,P_{f_{n}}\right\}$ and entry points $\left\{P_{e_{1}},P_{e_{2}},\ldots ,P_{e_{m}}\right\}$ . Each iteration involves predicting future positions, evaluating potential paths, and selecting the optimal path based on a cost function.

Given the real-time requirements of clinical interventions, particularly in procedures involving organ motion such as prostate treatments, the response time of the algorithm is crucial. The time complexity of the algorithm per iteration can be approximated as O(m×n) , where m is the number of entry points and n is the number of active targets. This complexity arises because each entry point is evaluated against each predicted target position to compute the associated costs R , D , and E .

Despite the dependence on the number of target points, the timing of this approach does not pose a problem for the latency requirements for several reasons:

1. Predictive Modeling Efficiency: The predictive model f used in the algorithm is designed for quick execution, leveraging recent advances in real-time data processing and machine learning. This ensures that the prediction step does not become a bottleneck.
2. Parallel Processing Capabilities: Modern clinical systems equipped with parallel processing capabilities can handle the computations for multiple paths simultaneously, significantly reducing the real-time computational burden.
3. Greedy Selection Strategy: The greedy approach to selecting the best entry point by minimizing the total cost $C_{j}$ ensures that once a path is deemed optimal at a given step, no further extensive calculations are needed for that iteration, enhancing responsiveness.

Therefore, although the timing analysis indicates a linear dependency on the number of targets, the impact on latency is mitigated by the efficiency of the predictive model, the capability for parallel processing, and the nature of the greedy selection algorithm. These factors ensure that the algorithm meets the stringent latency requirements of real-time medical procedures.

## 3 Results

### 3.1 Training and testing environment

#### 3.1.1 Dataset description

**FIGURE 10:**
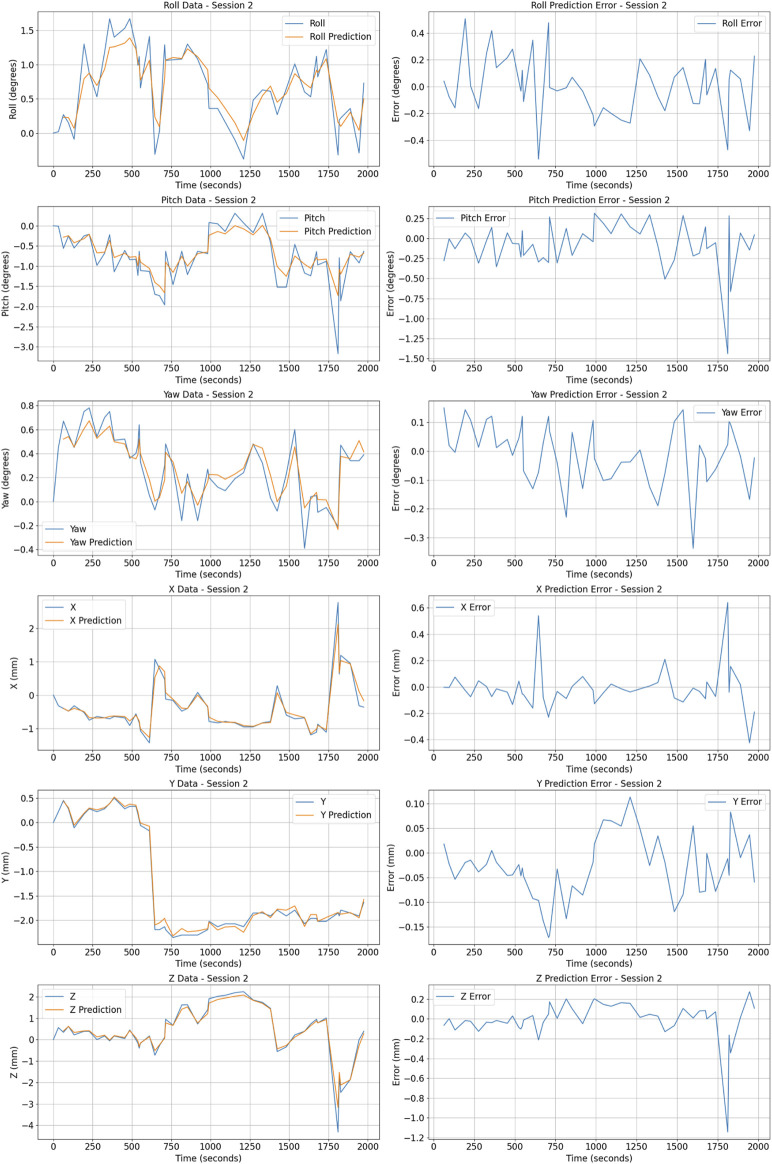
Posture prediction and error metrics for patient 1 Session 2. The figure shows actual (blue) vs predicted (orange) values for Roll, Pitch, Yaw (in degrees), and X, Y, Z coordinates (in millimeters) on the left. Prediction errors for these metrics are displayed on the right.

The dataset for training the Prostate Motion AI-based Prediction Model comprises around 42,822 motion data distributed on 162 patients undergoing radiotherapy. Each patient’s prostate motion was recorded over five (or six) sessions with approximately of 50 postures per session per patient, resulting in a comprehensive dataset that captures a wide array of movement patterns. The recorded data specifically includes the 3D prostate posture (See Figure 10, providing the spatial-temporal sequences necessary for the predictive modeling.

The distribution of the prostate posture include a mean distance from the origin computed for positional coordinates $\left(X_{c},Y_{c},Z_{c}\right)$ of 2.505mm , with a standard deviation of 2.070mm , ranging from 0.0 to 17.645mm . This provides an indication of the spatial spread relative to the origin.

The orientation data represented through angles $\left(Roll_{c},Pitch_{c},Yaw_{c}\right)$ shows distinct distributions. The mean roll angle is nearly zero −0.0018 with a standard deviation of 0.882, and it ranges between −3.8 and 4.0, indicating balanced rotational movements around the longitudinal axis. The pitch angle has a slight negative mean −0.132 , a standard deviation of 1.858, and exhibits a broader range from −10.01 to 9.44, reflecting more significant variability in tilt movements. Finally, the yaw angle, describing rotation around the vertical axis, has a mean of 0.046, a standard deviation of 0.813, and extends from −4.8 to 4.4, showing moderate variation in directional orientation.

These movements are due to: Firstly, respiration which leads to regular and predictable movements of the prostate. As the diaphragm contracts and relaxes, it causes the pelvic organs, including the prostate, to shift slightly both vertically and anteroposteriorly. This effect is generally consistent across sessions but can vary slightly between individuals based on their respiratory rate and depth.

Secondly, less predictable but natural movements are due to gastrointestinal activities, such as the passage of gas, which can cause transient shifts in the position of the pelvic organs. These movements are typically minor but must be accounted for in the predictive modeling of prostate motion.

Although patients are not under full anesthesia, their organ is fixed to remain as still as possible. Sudden movements are rare but can occur; however, these are typically quick and isolated incidents that do not significantly impact the overall dataset. The robustness of the AI model is designed to accommodate these rare deviations by focusing on the more predictable and consistent movement patterns driven by natural physiological processes.

#### 3.1.2 Hardware environment

The training and development of the CNN-LSTM model were conducted on a personal laptop. The specifications of the hardware environment are as follows:

• **Processor:** AMD Ryzen 7 7745HX,

• **Memory:** 32GB RAM,

• **Graphics Card:** NVIDIA RTX 4070.

#### 3.1.3 Software environment and trainning strategy

The software environment used for developing and training the CNN-LSTM model includes the following:

• **Programming Language:** Python 3.8

• **Deep Learning Library:** Keras

• **Visualization Tool:** Matplotlib

The dataset used for training the CNN-LSTM model consisted of patient data, which was split into training, validation, and test sets based on the number of patients. This approach ensures that the data from any single patient is only present in one of the sets.

• **Data Split:** The data was divided as follows:

• Training set: 70% of the patients

• Validation set: 10% of the patients

• Test set: 20% of the patients

### 3.2 Training and validation performance

The model’s performance was evaluated and compared to multiple baseline methods in Table 1 using Mean Squared Error (MSE) as the loss function, which quantifies the average squared difference between the estimated values and the actual value. The CNN-LSTM model achieved a best MSE of approximately 1.44, indicating a high level of accuracy in the prediction of the prostate’s center of gravity.

**TABLE 1:** Comparative table of various methods with MSE values.

Method	Mean squared error $\left(mm^{2}\right)$	Inference time (ms)
Linear Regression	5.19	**9**
Polynomial Regression	4.11	16
MLP	3.94	28
RNN	3.51	39
LSTM	2.37	41
**CNN-LSTM**	**1.44**	50

The bold values represent the best values for each column.

The complete training time amounted to approximately 1 min/epoch, this training phase involved multiple epochs of learning to ensure the model accurately captures the dynamic movements of the prostate across different sessions and patients.

Inference time, or the time it takes for the model to predict future prostate positions once trained, was optimized to meet the real-time requirements of therapeutic procedures. On average, the model achieved an inference time of 50 m per prediction. This rapid response rate ensure a real time performance in clinical settings (especially in MRI based environment).

**FIGURE 11:**
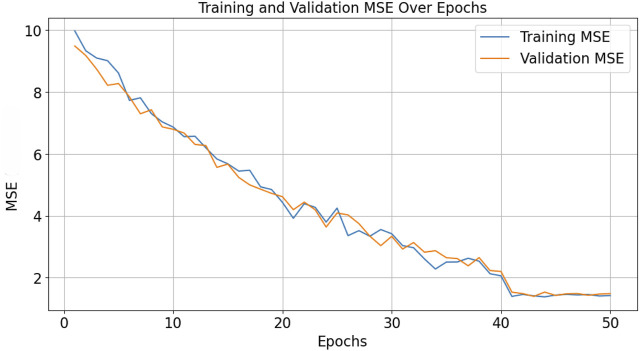
Training and validation MSE of the CNN-LSTM model.

The graph in Figure 11 illustrates the learning curve of the model, with the *x*-axis representing the number of epochs and the *y*-axis representing the MSE. The plot shows the trend of decreasing MSE as the number of epochs increases, both for training and validation datasets.

### 3.3 Adaptive optimisation result

We establish α as the base parameter with a value of 1, setting the reference scale for the total distance R between each entry point and its corresponding target point. To prioritize accuracy in reaching the predicted target positions, β is set to 1.5, emphasizing the importance of adhering to the predicted posture. λ is adjusted to match the order of magnitude of the distances, set to a value that equates its scale with that of the distances, approximately in the order of 100. This setting ensures a balanced influence of the number of entry points on the cost function, maintaining a proportionate impact alongside the distance components.

To effectively evaluate our approach, we utilize two straightforward metrics: the number of needles used during the procedure and the coverage percentage. The coverage percentage assess the treatment effectiveness. It is calculated as follows:

$$\text{Coverage}\;\text{Percentage}=\left(\frac{\text{Number}\;\text{of}\;\text{Target}\;\text{Points}\;\text{Successfully}\;\text{Reached}}{\text{Total}\;\text{Number}\;\text{of}\;\text{Target}\;\text{Points}}\right)\times 100\,\%$$ (10)

This formula provides a clear measure of how well the target areas are treated, reflecting the proportion of the intended treatment area that is actually reached by the end of the procedure. Comparing these metrics across different scenarios helps us evaluate the adaptive optimization algorithm compared to a traditional static approach.

**FIGURE 12:**
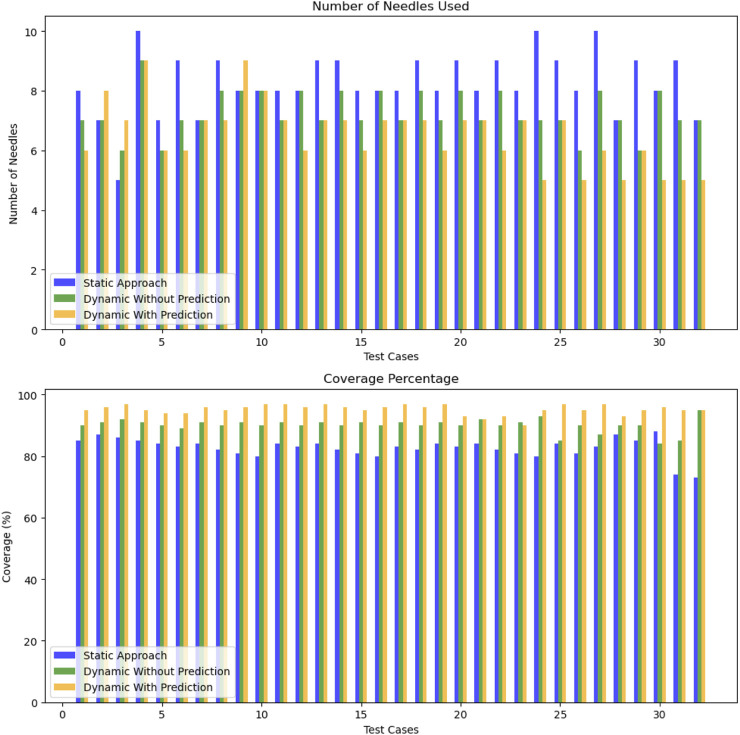
Performance comparaison between static and adaptive approaches.

The results of the adaptive optimization algorithm have been quantitatively assessed against both the traditional static approach and a dynamic method without prediction (where β=0 ) across ten different procedures (different movement scenarios from the patients dataset), as illustrated in Figure 12.

Our dynamic approach with prediction provides a slight reduction in the average number of needles 6.5 compared to 7.3 for the dynamic method without prediction and 8.25 for the static approach. Additionally, the average coverage performance for the dynamic method with prediction is around 95.25% , while the static approach marked an average performance of 90.06% , and the dynamic method without prediction achieved 82.7% . This improvement is due to the adaptive trait that reacts to the motion of the prostate and changes in position.

The significance of these results is supported by statistical testing. The *t*-test between the static approach and the dynamic approach with prediction yielded a t-statistic of 6.47 and a *p*-value of $8.75\times 10^{-9}$ , indicating a highly significant difference favoring the dynamic approach with prediction. Similarly, the *t*-test between the dynamic method without prediction and the dynamic method with prediction yielded a t-statistic of 3.26 and a *p*-value of $9\times 10^{-4}$ , again demonstrating a statistically significant improvement with the dynamic approach with prediction.

Overall, the dynamic approach demonstrates an average improvement of 12.72% , with some scenarios reaching up to 15.59% . Although in some instances the dynamic approach selects a higher number of needles compared to the static one, this leads to a significant gain in coverage of target points.

In summary, the adaptive optimization approach has proven to be more efficient and effective than both the static approach and the dynamic method without prediction. It ensures optimal needle usage and excellent coverage, which are critical for successful brachytherapy outcomes. Future work will focus on expanding the application of this approach to other procedural contexts and further refining the algorithm to enhance its decision-making capabilities.

## 4 Discussion

### 4.1 Key points and critics

The adaptive optimization approach demonstrates advancements over static methods, primarily due to its real-time predictive modeling and dynamic optimization, which have resulted in fewer needles used and high coverage.

The reduction in the number of needles used not only enhances patient comfort but is also indicative of the precision and efficiency of the predictive model. Fewer entry points reduce the risk of infection and tissue damage, which are critical considerations in clinical settings.

Furthermore, the high coverage rate achieved by our approach ensures that the treatment is comprehensive, addressing most if not all cancerous tissues effectively. This is particularly significant as it directly correlates with the success rate of the treatment.

Key points of the adaptive approach include:

• **Needle Efficiency:** Fewer needles are used in the dynamic approach, reducing patient discomfort and complication risks.
• **Target Coverage:** High coverage is consistently achieved, highlighting the method’s precision.
• **Procedural Advantages:** Resource utilization and potential reductions in procedure time suggest improved surgical workflow efficiency.

In terms of outlier scenarios where predictions are taken by surprise and the error margin is high, an expert intervention can be implemented to stop the procedure or deactivate the predictive approach thus potentially offering better management of such outliers compared to traditional methods.

The approach, however, is based on assumptions that merit further evaluation:

• **Prostate Movement:** The model’s restriction to translational and rotational movements may not capture all prostate behaviors.
• **Needle Impact:** The assumed negligible needle impact on prostate movement may vary between patients.
• **Unpredictable Movements:** The model does not currently account for abrupt, irregular movements.
• **Inference Time:** The model’s inference time could be further optimized for instantaneous surgical response.

In conclusion, the dynamic approach not only upholds the standards of traditional interventions but also introduces significant improvements in handling, efficiency, and outcomes, making it a valuable addition to the field of surgical interventions for prostate cancer.

### 4.2 Conclusion and future work

This research enhances prostate minimally invasive interventions by introducing a method that combines real-time movement data with predictive modeling to optimize the procedure. Unlike traditional approaches, our dynamic method with prediction reduces the average number of needle entry points to 6.5 from 8.25, improving procedural efficiency. The effectiveness of this method is demonstrated by its high coverage performance of 95.25% compared to the static method’s 82.7% and the dynamic method without prediction’s 90.06% . The dynamic method without prediction, while not as efficient as the predictive model, still shows significant improvements over the static approach, reducing needle entry points to 7.3 and achieving a coverage performance of 90.06% .

A key component of this approach is an AI algorithm that uses data to predict prostate positions enabling real time adjustments to the plan for better accuracy and fewer needle insertions. Additionally incorporating the CoBra template grid optimizes needle placement. Streamlines the process.

In summary this dynamic optimization strategy represents an advancement in path planning greatly improving treatment outcomes and paving the way, for wider clinical use.

Future efforts should focus on refining this optimization algorithm and integrating more robust predictive models specifically integrating the needle impact on the prostate posture as well as some basic deformations to further improve the efficacy of medical treatments in precision-critical scenarios.

## Data Availability

The data analyzed in this study is subject to the following licenses/restrictions: Patients prostate movements dataset provided by CHU Oscar Lambert with restricted usage. Requests to access these datasets should be directed to m-ledeley@o-lambret.fr.

## References

[B1] Bai S. Kolter J. Z. Koltun V. (2018). An empirical evaluation of generic convolutional and recurrent networks for sequence modeling. arXiv preprint arXiv:1803.01271.

[B2] Belarouci A. Dhaliwal S. S. Sanz-Lopez M. Verbrugghe F. Lakhal O. Chettibi T. (2022). Cooperative brachytherapy robotic concept for localized cancer treatment under real-time mri. IEEE Trans. Med. Robotics Bionics 4, 667–681. 10.1109/tmrb.2022.3185796

[B3] Bengs M. Sprenger J. Gerlach S. Neidhardt M. Schlaefer A. (2023). Real-time motion analysis with 4d deep learning for ultrasound-guided radiotherapy. IEEE Trans. Biomed. Eng. 70, 2690–2699. 10.1109/TBME.2023.3262422 37030809

[B4] Bray F. Laversanne M. Sung H. Ferlay J. Siegel R. L. Soerjomataram I. (2024). Global cancer statistics 2022: globocan estimates of incidence and mortality worldwide for 36 cancers in 185 countries. CA A Cancer J. Clin. 74, 229–263. 10.3322/caac.21834 38572751

[B5] Chauhan A. Vig L. (2015). “Anomaly detection in ecg time signals via deep long short-term memory networks,” in 2015 IEEE International Conference on Data Science and Advanced Analytics (DSAA), Paris, France, 19-21 October 2015 (IEEE), 1–7.

[B6] Chrystall D. Mylonas A. Hewson E. Martin J. Keall P. Booth J. (2023). Deep learning enables mv-based real-time image guided radiation therapy for prostate cancer patients. Phys. Med. Biol. 68, 095016. 10.1088/1361-6560/acc77c 36963116

[B7] Comelli A. Dahiya N. Stefano A. Vernuccio F. Portoghese M. Cutaia G. (2021). Deep learning-based methods for prostate segmentation in magnetic resonance imaging. Appl. Sci. Basel, Switz. 11, 782. 10.3390/app11020782 PMC7932306 33680505

[B8] Dhaliwal S. S. Chettibi T. Belarouci A. Dherbomez G. Coelen V. Merzouki R. (2019). “Cooperative brachytherapy for prostate cancer under mri guidance,” in 2019 Fifth International Conference on Advances in Biomedical Engineering (ICABME), Tripoli, Lebanon, 17-19 October 2019 (IEEE), 1–4.

[B9] Dhaliwal S. S. Chettibi T. Wilby S. Polak W. Palmer A. L. Reynaert N. (2021a). Review of clinical and technological consideration for mri-guided robotic prostate brachytherapy. IEEE Trans. Med. Robotics Bionics 3 (3), 583–605. 10.1109/tmrb.2021.3097127

[B10] Dhaliwal S. S. Wilby S. Firouzy S. Boni K. B. de Vries M. Navarro S. E. (2021b). “Cobra robot for localized cancer treatment and diagnosis under real-time mri,” in AUTOMED 2021-Interdisciplinary Symposium Automation in Medical Engineering.

[B11] Ernst T. Armstrong B. S. R. Edmund P. T. (2019). Motion tracking system and method for real time adaptive imaging and spectroscopy.

[B12] Frohwein L. J. Büther F. Schäfers K. P. (2019). Estimation of physiological motion using highly accelerated continuous 2d mri. arXiv preprint arXiv:1905.08176.

[B13] Henry A. Pieters B. R. André Siebert F. Hoskin P. UROGEC group of GEC ESTRO with endorsement by the European Association of Urology (2022). GEC-ESTRO ACROP prostate brachytherapy guidelines. Radiother. Oncol. 167, 244–251. 10.1016/j.radonc.2021.12.047 34999134

[B14] Huynh T. T. Nguyen M. H. Nguyen T. Nguyen P. L. Weidlich M. Nguyen Q. V. H. (2022). Efficient integration of multi-order dynamics and internal dynamics in stock movement prediction. United States: ACM.

[B15] Hyuck L. J. Jin H. W. Mo Y. H. Seung K. H. (2020). Surgery optimization method and device.

[B16] Ismail M. Sengur A. (2021). Deep learning-based time series forecasting: an experimental evaluation. Neural Comput. Appl. 33, 417–432. 10.1016/j.eswa.2020.114054

[B17] Kannan S. Teo B.-K. K. Solberg T. D. Hill-Kayser C. E. (2017). Organ motion in pediatric high-risk neuroblastoma patients using four-dimensional computed tomography. J. Appl. Clin. Med. Phys. 18, 107–114. 10.1002/ACM2.12012 28291918 PMC5689899

[B18] Kavak A. G. Surucu M. Ahn K.-H. Pearson E. Aydogan B. (2022). Impact of respiratory motion on lung dose during total marrow irradiation. Front. Oncol. 12, 924961. 10.3389/fonc.2022.924961 36330489 PMC9622752

[B19] Keerthana M. Reddy D. G. V. R. Maruthiram M. K. B. (2023). Landmark tracking in liver us images using cascade convolutional neural networks with long short-term memory. Int. J. Res. Appl. Sci. Eng. Technol. (IJRASET) 11, 2456–2465. 10.22214/ijraset.2023.55895 PMC9893725 36743834

[B20] Krebs J. Mansi T. Ayache N. Delingette H. (2019). Probabilistic motion modeling from medical image sequences: application to cardiac cine-mri. arXiv preprint arXiv:1907.13524.

[B21] Lakhal O. Chettibi T. Belarouci A. Youcef-Toumi K. Merzouki R. (2023). Optimisation of path planning for minimally invasive interventions on prostate using mr-robot: application to on-live pets. IFAC-PapersOnLine 56, 11621–11626. 10.1016/j.ifacol.2023.10.484

[B22] Li D. Zhong W. Deh K. M. Nguyen T. D. Prince M. R. Wang Y. (2019). Discontinuity preserving liver mr registration with three-dimensional active contour motion segmentation. IEEE Trans. Biomed. Eng. 66, 1884–1897. 10.1109/TBME.2018.2880733 PMC6565504 30418878

[B23] Li Y. Yang C. Bahl A. Persad R. Melhuish C. (2022). A review on the techniques used in prostate brachytherapy. Cognitive Comput. Syst. 4, 317–328. 10.1049/ccs2.12067

[B24] Liao L. Hu Z. Hsu C. Y. Su J. (2023). Fourier graph convolution network for time series prediction. Mathematics 11, 1649. 10.3390/math11071649

[B25] Morid M. A. Sheng O. R. L. Dunbar J. (2021). Time series prediction using deep learning methods in healthcare. arXiv preprint arXiv:2108.13461.

[B26] National Comprehensive Cancer Network (2024). Nccn clinical practice guidelines in oncology: prostate cancer. United States: National Comprehensive Cancer Network. Available at: https://www.nccn.org/professionals/physician_gls/pdf/prostate.pdf (Accessed April 4, 2024). 10.6004/jnccn.2010.0012 20141676

[B27] Ng J. Gregucci F. Pennell R. Nagar H. Golden E. B. Knisely J. P. (2023). Mri-linac: a transformative technology in radiation oncology. Front. Oncol. 13, 1117874. 10.3389/fonc.2023.1117874 36776309 PMC9911688

[B28] Purushotham S. Meng C. Che Z. Liu Y. (2019). Benchmark of deep learning models on large healthcare mimic datasets. arXiv preprint arXiv:1710.08531. 10.1016/j.jbi.2018.04.007 29879470

[B29] Ruijters D. (2021). Artificial intelligence in minimally invasive interventional treatment. arXiv: Computer Vision and Pattern Recognition.

[B30] Senneville B. D. D. Hamidi A. E. Moonen C. T. W. (2015). A direct pca-based approach for real-time description of physiological organ deformations. IEEE Trans. Med. Imaging 34, 974–982. 10.1109/TMI.2014.2371995 25423649

[B31] Su H. Li S. Manivannan J. Bascetta L. Ferrigno G. Momi E. D. (2019). “Manipulability optimization control of a serial redundant robot for robot-assisted minimally invasive surgery,” in 2019 International Conference on Robotics and Automation (ICRA), Montreal, QC, Canada, 20-24 May 2019 (IEEE), 1323–1328.

[B32] Taillez A. Bimbai A.-M. Lacornerie T. Le Deley M.-C. Lartigau E. F. Pasquier D. (2021). Studies of intra-fraction prostate motion during stereotactic irradiation in first irradiation and re-irradiation. Front. Oncol. 11, 690422. 10.3389/fonc.2021.690422 34336678 PMC8316636

[B33] Vijayan S. Klein S. Hofstad E. F. Lindseth F. Ystgaard B. Langø T. (2014). Motion tracking in the liver: validation of a method based on 4d ultrasound using a nonrigid registration technique. Med. Phys. 41, 082903. 10.1118/1.4890091 25086560

[B34] Wang Z. Zhou F. Trajcevski G. Zhang K. Zhong T. (2023). Learning dynamic temporal relations with continuous graph for multivariate time series forecasting. Proc. AAAI Conf. Artif. Intell. 37, 16358–16359. 10.1609/aaai.v37i13.27039

[B35] Xiao H. Huang Y. Pan Z. Li W. Hu Y. Lin G. (2023). Multi-task time series forecasting based on graph neural networks. Entropy 25, 1136. 10.3390/e25081136 37628166 PMC10453913

[B36] Yang X. Zhang S. Zhang X. Wu D. Su J. (2023). Machine learning and deep learning based time series prediction and forecasting of ten nations’ COVID-19 pandemic. SN Comput. Sci. 4. 10.1007/s42979-022-01493-3 PMC9748400 36532634

