# Supplementary material for: Adaptive approach for tracking movements of biological targets: application to robot-based intervention for prostate cancer
Source: Front Robot AI. 2024 Aug 12;11:1416662. doi: 10.3389/frobt.2024.1416662 (PMC11345532; doi:10.3389/frobt.2024.1416662)
Supplement: Supplementary file 1 [file DataSheet1.PDF]

Supplementary Material

1 COBRA ROBOT KINEMATICS

This section develop the CoBra robot Inverse Kinematic Model (IKM), is a nonlinear function f_{IKM}, allowing to describe the position of the finite target points (needle tip or Tool Centre Point - TCP) (x_t, y_t, z_t) to the 5 joint positions of the robot $(L_{f_1}, L_{f_2}, L_{r_1}, L_{r_2}, L_g)$, as follows:

$$[L_{f1}, L_{f2}, L_{r1}, L_{r2}, L_g] = f_{IKM}(x_t, y_t, z_t) \tag{S1}$$

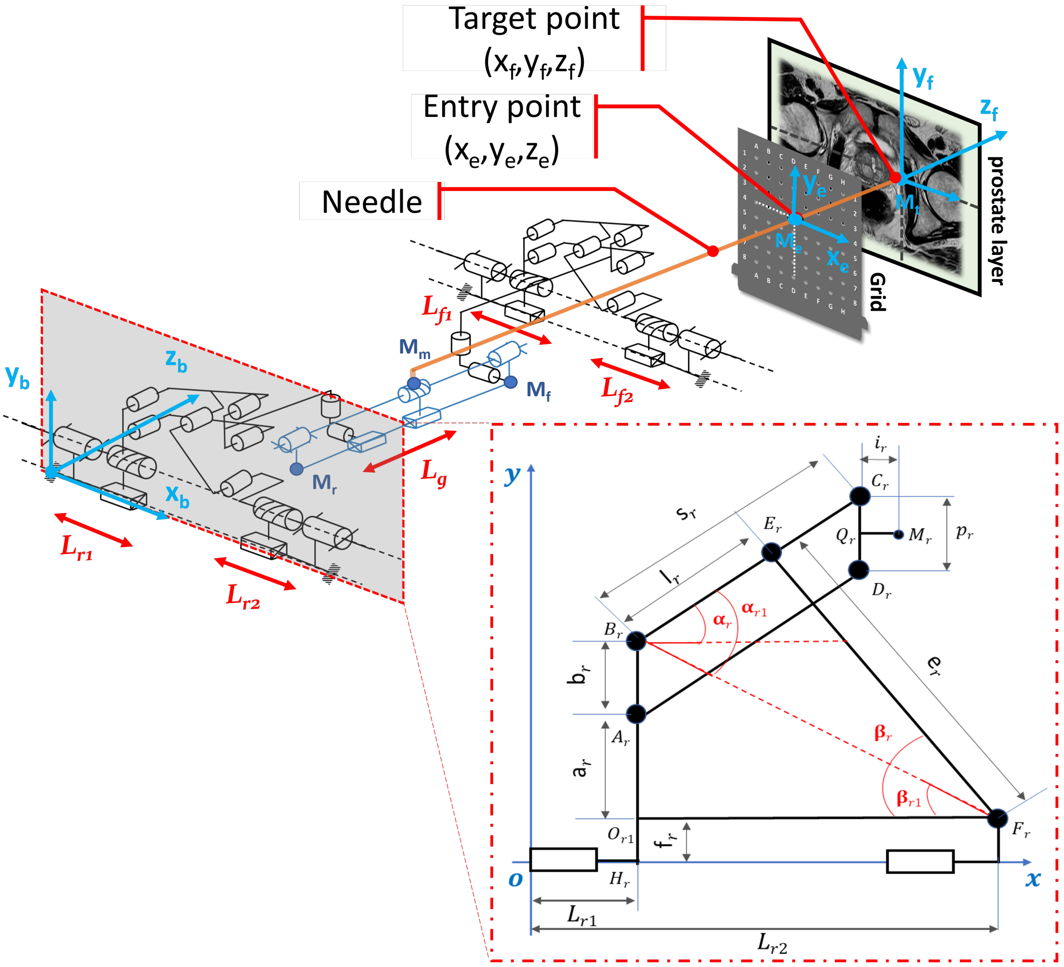

Figure S1. Robot kinematics of the CoBra Robot

The position of the target point can be reached with a multitude of possibilities depending on the orientation of the needle. The input grid prevents the needle from bending during insertion, but on the other hand, it adds constraints to the robot kinematics. Indeed, after insertion, the orientation of the needle is constrained throughout the robot's movement. This also implies a finite and reduced number of possibilities to reach a target point due to the joint position limitations. The kinematic model is required to control the needle tip but also to verify beforehand the accessibility of the needle tip according to the constraint.

Let a p a target point be defined by the transformation matrix ${}^{t}_{b}T$ expressed in base frame $\Re_b\{x_b, y_b, z_b\}$ of the robot, Fig. S1.

$$
{}^{t}_{b}T = \begin{bmatrix} u_1 & v_1 & w_1 & x_t \\ u_2 & v_2 & w_2 & y_t \\ u_3 & v_3 & w_3 & z_t \\ 0 & 0 & 0 & 1 \end{bmatrix} \tag{S2}
$$

Knowing the desired entry point and target point, it is therefore possible to determine the directional vectors. **u**, **v** and **w** of the reference frame attached to the point M_f along the direction M_rM_f. Based on the coordinates of the entry point $\{x_e, y_e, z_e\}$ and the target point $\{x_t, y_t, z_t\}$, the vector **w** can be obtained as follows:

$$
\mathbf{w} = \frac{[x_t - x_e\ ,\ y_t - y_e\ ,\ z_t - z_e]^T}{\sqrt{(x_t - x_e)^2 + (y_t - y_e)^2 + (z_t - z_e)^2}} \tag{S3}
$$

The vector **u** is normal to the vector **w** and is parallel to the plane ZX

$$
\mathbf{u} = \frac{[w(3)\ ,\ 0\ ,\ \ -w(1)]^T}{\sqrt{(w(3))^2 + (-w(1))^2}} \tag{S4}
$$

The vector **v** is perpendicular to the plane formed by the vectors **u** and **w**

$$
\mathbf{v} = \mathbf{w} \times \mathbf{u} \tag{S5}
$$

Where $\times$ represents the vector product.

The rotation matrix of the matrix ${}^{t}_{b}T$ can thus be obtained. The constraint of the input grid makes the needle less flexible as it applies a supporting force, so we can assume that the needle can be considered as a rigid tube. Thus, we can relate the coordinates of the needle tip to the point M_f of the CoBra guide robot, knowing the dimensions of the needle.

$$
\begin{aligned}
{}^{t}_{b}T &= {}^{Mf}_{b}T\,{}^{Mm}_{Mf}T\,{}^{t}_{Mm}T \\
{}^{Mf}_{b}T &= {}^{t}_{b}T\left({}^{t}_{Mm}T\right)^{-1}\left({}^{Mm}_{Mf}T\right)^{-1}
\end{aligned} \tag{S6}
$$

The expression of L_g can therefore be deduced

$$L_g = \frac{g + g_0 - {}^{Mf}_{b}T(3,4)}{{}^{Mf}_{b}T(3,3)} \tag{S7}$$

Where g and g_0 are the geometric parameters of the robot. Given the value of L_g, it is therefore possible to obtain the transformation matrix

$$ {}^{Mr}_{b}T =^{Mf}_{b} T^{Mr}_{Mf}T(L_g) \tag{S8}$$

From the transformation matrix ${}^{Mr}_{b}T$ and ${}^{Mf}_{b}T$ represent the transformation matrix of the points M_r and M_f, respectively, relative to the base frame of the robot, the values of the joint positions L_{f1}, L_{f2}, L_{r1} and L_{r2} can be deduced .

$$\begin{aligned} L_{f1} &= {}^{Mf}_{b}T(1,4) - d_1.cos(\alpha_1) - i_1; \\ L_{f2} &= L_{f1} + c_1.cos(\alpha_1) + e_1.cos(\beta_1) \end{aligned} \tag{S9}$$

Where : $\alpha_1 = \arcsin\left(\frac{{}^{Mf}_{b}T(2,4) - (a_1 + b_1/2 + f_1)}{d_1}\right)$ and $\beta_1 = \left(\frac{a_1 + b_1 + c_1.\sin(\alpha_1)}{e_1}\right)$

The same applies to the rear part of the robot :

$$\begin{aligned} L_{r1} &= {}^{Mr}_{b}T(1,4) - d_2.cos(\alpha_2) - i_2; \\ L_{r2} &= L_{r1} + c_2.cos(\alpha_2) + e_2.cos(\beta_2) \end{aligned} \tag{S10}$$

Where : $\alpha_2 = \arcsin\left(\frac{{}^{Mr}_{b}T(2,4) - (a_2 + b_2/2 + f_2)}{d_2}\right)$ and $\beta_2 = \left(\frac{a_2 + b_2 + c_2.\sin(\alpha)}{e_2}\right)$

a_1, a_2,b_1,b_2,c_1, c_2,e_1,e_2,f_1,f_2,i_1 and i_2 represent the geometric parameters of the robot, as illustrated in Fig. S1.
